# Netrin-1 attenuates the progression of renal dysfunction by blocking endothelial-to-mesenchymal transition in the 5/6 nephrectomy rat model

**DOI:** 10.1186/s12882-016-0260-4

**Published:** 2016-05-12

**Authors:** Jiuxu Bai, Junfeng Hao, Xiaoling Zhang, Hanmin Cui, Jingming Han, Ning Cao

**Affiliations:** Department of Blood Purification, General Hospital of Shenyang Military Area Command, No.83, Wenhua Road, Shenyang, 110016 Liaoning Province China

**Keywords:** Netrin-1, Endothelial-to-mesenchymal transition, Renal interstitial fibrosis, 5/6 Nephrectomized rats

## Abstract

**Background:**

Endothelial-to-mesenchymal transition (EndoMT) is a crucial event during kidney interstitial fibrosis and it is believed to be inhibited by netrin-1. Our aim was to determine the influence of netrin-1 on renal EndoMT in chronic kidney disease by studying its effect in 5/6 nephrectomized (Nx) rats.

**Methods:**

Male Sprague–Dawley rats were divided into three groups (10 rats/group): sham-operated rats treated with control adenovirus; 5/6 Nx rats treated with control adenovirus; and 5/6 Nx rats treated with recombinant adenovirus expressing the netrin-1 gene (Ad-netrin-1). Rats were sacrificed 13 weeks after surgery. Blood urea nitrogen (BUN) and serum creatinine (Scr) levels were measured regularly after surgery. After the rats were sacrificed, pathological changes in renal tissues were analyzed histologically. Immunofluorescence was performed to evaluate the co-expression of CD31 and α-SMA. CD31, α-SMA and Snail mRNA were detected by RT-PCR. Protein expression was detected by western blot.

**Results:**

Renal function and histopathological damage were significantly improved in Ad-netrin-1-treated 5/6 Nx rats. In the sham and control-treated 5/6 Nx rats, the percentage of CD31^+^/α-SMA^+^ cells increased, which indicated EndoMT. However, the percentage of CD31^+^/α-SMA^+^ cells were reduced in the netrin-1-treated 5/6 Nx rats, which indicates netrin-1-induced blocking of EndoMT.

**Conclusion:**

From the results, it seems that netrin-1 attenuates the progression of renal dysfunction by inhibiting EndoMT in 5/6 Nx rats. Netrin-1 can therefore be considered as a potential therapeutic agent for the treatment of renal fibrosis.

## Background

Interstitial fibrosis has long been viewed as a common feature of chronic kidney disease (CKD), and it is a characteristic hallmark that indicates the prognosis of any kind of progressive kidney disease. Interstitial fibrosis may occur diffusely, with or without atrophic tubules, or focally, in association with atrophic tubules. The transition of renal epithelial cells to myofibroblasts in renal fibrosis has been intensively investigated, and increasing evidence suggests that the contribution of epithelial-mesenchymal transition (EMT) to the pool of activated fibroblasts is responsible for renal interstitial fibrosis in several experimental models [1–3].

Endothelial-mesenchymal transition (EndoMT) has emerged as another potentially important mechanism that is involved in both the developmental and pathological processes of kidney interstitial fibrosis. EndoMT is a complex process via which certain endothelial cells lose their endothelial characteristics and transform into mesenchymal or smooth muscle cells (SMCs) [4]. Fibroblasts are likely to be of endothelial origin, so it is possible that EndoMT contributes substantially to the accumulation of fibroblasts in the development and progression of renal fibrosis. EndoMT was first investigated as a critical process in heart development, and studies have shown that EndoMT contributes to the development of diabetic renal interstitial fibrosis, diabetic nephropathy, and cardiac fibrosis, which indicates a relationship between EndoMT and fibrosis [5–7] . Moreover, a recent study has demonstrated that EndoMT can contribute to the progression of multiple diseases in mouse models of CKD [8]. Further, EndoMT is known to contribute to the accumulation of activated fibroblasts and myofibroblasts in fibrotic kidneys [8]. However, the mechanism via which EndoMT affects fibrosis remains largely unknown.

Netrin-1 is a laminin-related secreted protein that is widely expressed in many tissues, including renal tissues. In recent studies, netrin-1 was shown to play a role in the migration of vascular endothelial cells and accelerating angiogenesis [9, 10], tumor progression, and growth and regulation of inflammation [11–13]. In particular, dysregulation of netrin-1 after ischemia contributes to the development of renal failure; further, studies indicate that downregulation of netrin-1 in vascular endothelial cells may promote endothelial cell activation and infiltration of leukocytes into the kidney, thereby enhancing tubular injury [14]. Netrin-1 is also known to regulate inflammatory cell migration and their functions in many diseases and suppress acute kidney injury (AKI) [15]. However, whether netrin-1 is associated with the anti-EndoMT mechanisms in CKDs is still unknown.

In this study, we investigated whether EndoMT occurs in 5/6 nephrectomized (Nx) rats and whether it contributes to the development of renal interstitial fibrosis. This model is often used to study the mechanisms of and potential therapeutic approaches to progression of CKD with renal reduction [16]. Meanwhile, we assessed the effect of netrin-1 on renal EndoMT in order to determine whether it provides protection against renal dysfunction in 5/6 Nx rats.

## Methods

### Construction of a recombinant netrin-1-expressing adenovirus

A netrin-1-expressing adenovirus was created using the AdEasy Vector system (Qbiogene, Nottingham, UK) as previously described [17] . The *Pac*I-linearized recombinant plasmid was then transfected into HEK293 cells (ATCC, Manassas, VA, USA). Adenovirus titers were determined by a plaque-forming assay, and expressed as the number of plaque-forming units (PFU). Virus stocks were amplified by culturing HEK293 cells with low-passage virus stocks, and amplification was continued until the titer reached 10^10^ PFU/ml. Aliquots of recombinant adenoviruses were then frozen at−80 °C until further use.

### Animals and experimental protocol

All animal procedures were conducted according to the animal care and ethics laws and were approved by the Animal Care Committee of the General Hospital of Shenyang Military Area Command. The 5/6 Nx rat model of chronic renal failure was established according to the method published by Ghosh et al. [18]. Thirty male Sprague–Dawley rats (8–10 weeks old, weighing 250–300 g) were obtained from the General Hospital of Shenyang Military Area Command Animal Centre (Shenyang, China), and maintained at 22 ± 1 °C, with relative air humidity at 60 ± 5 % and with free access to food and water. They were then randomly divided into one of three groups (*n* = 10 rats/group): sham-operated rats treated with the control adenovirus (empty vector); 5/6 Nx rats treated with the control adenovirus (empty vector); and 5/6 Nx rats treated with the netrin-1-expressing recombinant adenovirus (Ad-netrin-1). The rats in the sham-operated group were anesthetized by intraperitoneal injection of 10 % chloral hydrate (3 ml/kg) followed by surgical exposure and removal of the renal capsule. The 5/6 Nx rats first underwent a partial left nephrectomy (removal of 1/3 of the superior pole and 1/3 of the inferior pole) under anesthesia. The animals were then returned to the vivarium to recover. One week later, they underwent a total right nephrectomy, thus accomplishing a 5/6 nephrectomy. During the second surgery, the 5/6 Nx rats were injected with 1 × 10^8^ PFU of control adenovirus (empty vector) or Ad-netrin-1 via the vena caudalis as reported in previous studies [19]. All animals were sacrificed at the 13th week of the experiment, by an intraperitoneal injection of sodium pentobarbital (50 mg/kg). Blood and kidney tissues were immediately collected and kept at−80 °C for further analysis.

### Biochemical analyses

Blood samples were collected from all animals at weeks 12 after surgery, for analysis of blood urea nitrogen (BUN) and serum creatinine (Scr). BUN and Scr concentrations were assayed with an Olympus 400 clinical chemistry analyzer.

Renal tissues were fixed in 4 % buffered paraformaldehyde and then embedded in paraffin. Then, 4-μm thick sections were generated and stained with hematoxylin and eosin (H&E) and Masson’s trichrome. The H&E-stained slices were examined by optical microscopy for pathological changes, and the slides stained with Masson’s trichrome were analyzed for interstitial fibrosis as previously described [20]. To determine the percentage of interstitial space in the kidney, five consecutive fields were randomly selected in the renal cortex and evaluated under a 400× lens on a 10 × 10 grid-imprinted reticule. All points not counted within tubular cells, lumen, glomerulus, or vascular spaces were considered as interstitial points. This fraction represented the relative interstitial volume. Results were expressed as percentages of the measured area, representing the interstitial space and being the relative volume of the interstitium.

### Immunofluorescence analysis

Frozen tissues were cut into 4-μm thick sections and fixed in 100 % acetone at−20 °C for 10 min. After washing with phosphate-buffered saline and blocking with 5 % bovine serum albumin (BSA) for 1 h, the tissues were incubated with primary antibodies specific for CD31 (Santa Cruz Biotechnology, Europe) and α-SMA (Abcam, England) at 4 °C overnight. The tissues were then incubated with a mixture of two secondary antibodies that had been raised in different species and conjugated to different fluorochromes (fluorescein isothiocyanate-conjugated goat anti-mouse and Cy3-conjugated goat anti-rabbit), and were incubated in 1 % BSA for 1 h at room temperature in the dark. For the negative control experiments, the primary antibody was replaced with non-immune IgG, which provided minimal labeling. Images were captured using an LSM5 Image Browser (Zeiss) and analyzed using a laser scanning confocal microscope (LSM 510 META, Zeiss). The areas showing CD31 and α-SMA immunoreaction were measured, and fluorescence intensity was analyzed as previously described [21].

### Real-time PCR

Frozen tissue samples were lysed in TRIzol reagent (Invitrogen, Carlsbad, CA), and total RNA was extracted according to the manufacturer’s instructions. The levels of transcripts were determined by real-time PCR (RT-PCR) using SYBR ®Premix Ex Taq ™ II (TaKaRa, Shiga, Japan) on an ABI 7901HT series PCR cycler (Applied Biosystems, Foster City, CA, USA). The data were normalized to β-actin expression and further normalized to the negative control. Primers were obtained from Sangon Biological Engineering Technology and Services (Shanghai, China), and the following specific primers were designed: β-actin, 5′-CCCACTCTTCCACCT TTG-3′ (sense) and 5′-TAGCCATATTCATTGTCATACC-3′ (antisense); CD31, 5′-CTCCATCCTGTCGGGTAA-3′ (sense) and 5′-TCATTCACGGTTTCTTCG-3′ (antisense); α-SMA, 5′- GGCAACCTCAAGAAGTCCC−3′ (sense) and 5′- GTGCAGCCATCCACAAGC−3′ (antisense); Snail,5′-GGCTGATGGAAGGCAGAG−3′ (sense) and 5′- CCAGTGGGTTGGCTTTAGTT−3′ (antisense).

### Western blotting

Total protein was extracted from tissue samples with a commercial kit (GE healthcare Biosciences, Pittsburg, PA, USA). The protein concentrations in the lysates were assayed by the BCA assay using commercial kits (Invitrogen). According to the assay results, the lysates were adjusted to a protein concentration of 1 g/l and kept in Eppendorf tubes (35 μl/tube) at−70 °C before subsequent use. The protein (40 μg) obtained from each lysate was electrophoresed in a gel containing 8 % sodium dodecyl sulfate. The separated fractions were transferred to a nitrocellulose membrane, treated with skimmed milk for 2 h to block non-specific antibody-binding sites, rinsed with Tris-buffered saline (TBS), and incubated overnight at 4 °C with primary antibodies specific to Snail (1:1000; Cell Signaling Technology, Beverly, Massachusetts), CD31 (1:1000; Santa Cruz Biotechnology, Europe) and α-SMA (1:500; Abcam, England). After rinsing with TBS (10 min, three times each), the membrane was incubated with a horseradish peroxidase-labeled secondary antibody (1:5000; Sigma-Aldrich Co.) at room temperature for 1 h, rinsed with TBST (Tris-buffered saline with 10 % Tween-20) and developed using the enhanced chemiluminescence kit (ECL, GE Healthcare Biosciences). Equal protein loading was confirmed by immunostaining against β-actin (1:4000; Sigma-Aldrich Co). The membrane was imaged and analyzed with a Multimage Light Cabinet (Alpha Innotech Corp., San Leandro, California).

### ELISA studies TGF-β

Transforming growth factor (TGF)-β serum levels were determined by ELISA (R&D Systems) according to the manufacturer’s instructions. ELISA results were read on a Fusion from PerkinElmer (Boston, MA).

### Statistical analyses

The data were analyzed by analysis of variance (ANOVA) using the SPSS 15.0 (SPSS, Chicago, IL, USA) software and are expressed as mean ± standard deviation (SD). The results were considered significant at *P* < 0.05.

## Results

### Netrin-1 attenuates the progression of renal dysfunction in 5/6 Nx rats

Our previous studies showed that the 5/6 Nx group had substantially reduced netrin-1 expression compared with the sham-operated group, and that netrin-1 was not detectable in the interstitial space. The Ad-netrin-1-treated 5/6 Nx group showed substantially increased netrin-1 expression compared with the 5/6 Nx group [17].

**Fig. 1:**
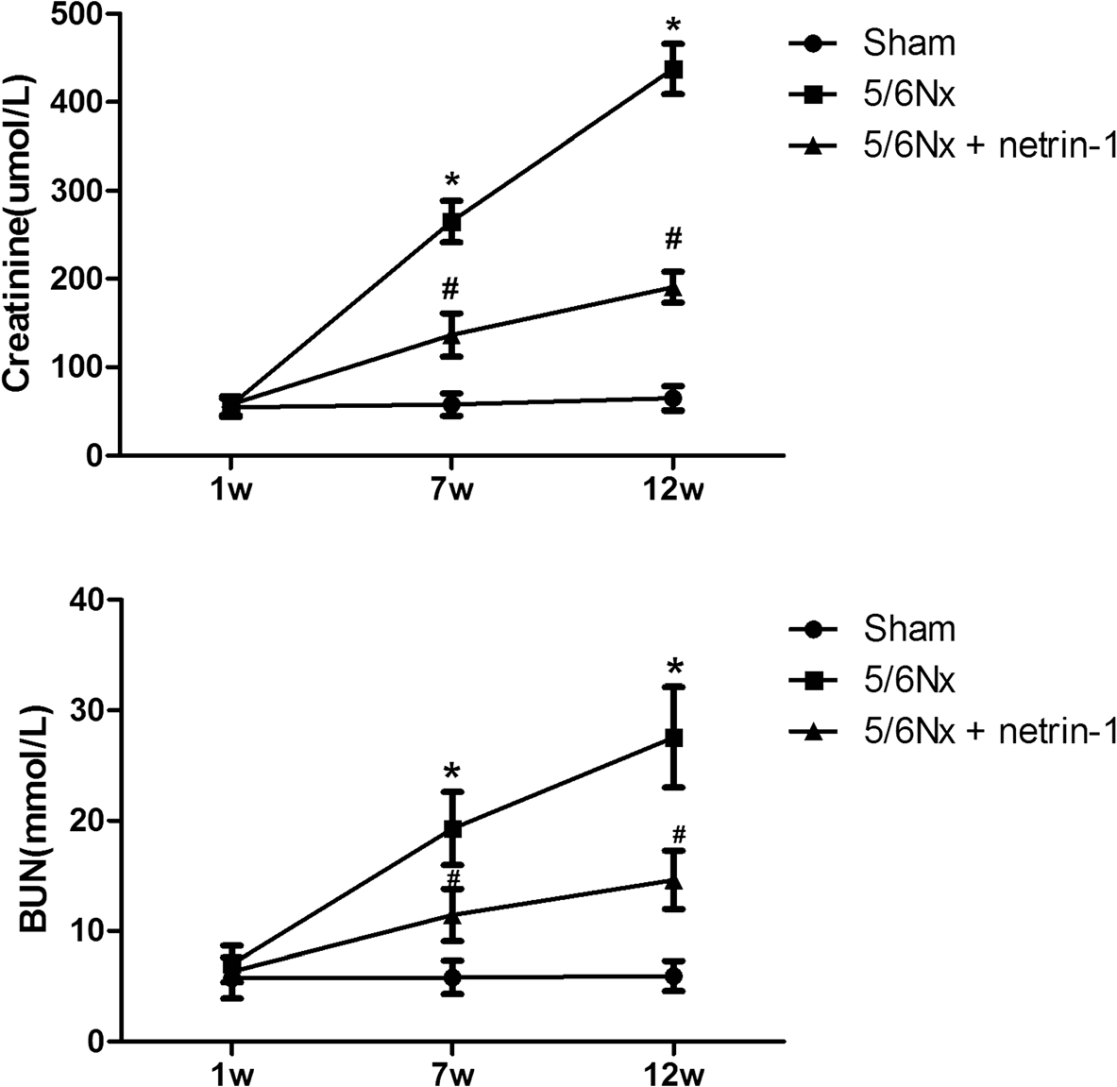
Changes in the blood urea nitrogen (BUN) and serum creatinine (Scr) levels in rats . From the seventh week after surgery, a significant increase in the Scr and BUN level was observed in the 5/6 Nx groups with and without Ad-netrin-1 treatment, in comparison with the sham-operated group. At postsurgery weeks 7 and 12, the rats that received Ad-netrin-1 had significantly lower BUN and Scr levels than the 5/6 Nx rats administered the control adenovirus. *, *P* < 0.05 vs. sham; #, *P* < 0.05 vs. 5/6 Nx

**Fig. 2:**
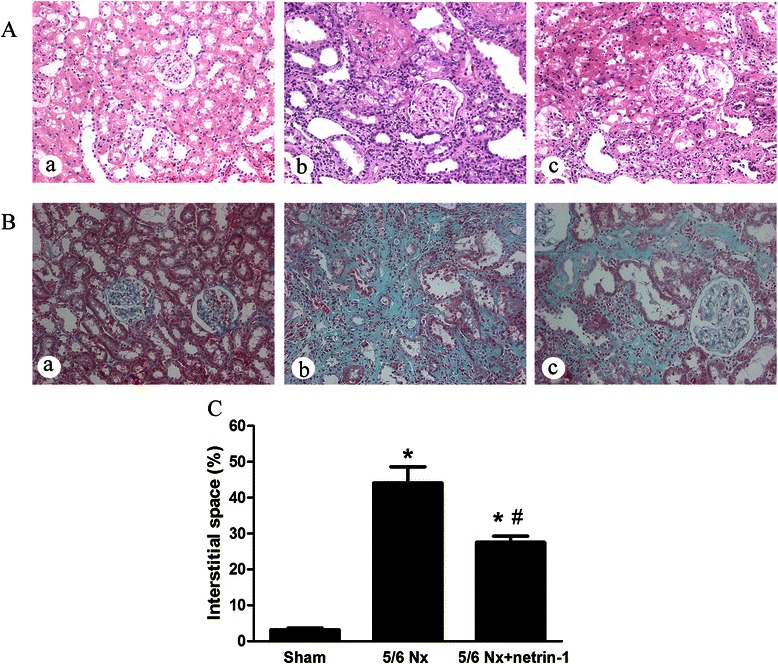
Histological findings at postoperative week 13. Hematoxylin and eosin staining **a** and Masson’s trichrome staining **b** in the renal tissue of sham-operated rats treated with control adenovirus (*a*), 5/6 Nx rats treated with control adenovirus (*b*) and 5/6 Nx rats treated with Ad-netrin-1 (*c*; original magnification: 400×). Quantification of the percentage of interstitial space **c** in the kidney is presented in a bar chart; the relative interstitial volume was quantified by morphometric analysis of Masson trichrome-stained kidney sections in each group; all results are expressed as mean ± SEM; *, *P* < 0.05 vs. sham. #, *P* < 0.05 vs. 5/6 Nx group; *n* = 10 for each group

To determine the role of netrin-1 in CKD, renal function was determined by measuring BUN and Scr in all three groups of rats. As expected, the 5/6 Nx rats (*n* = 10) had significant elevations of Scr and BUN. The BUN and Scr levels were significantly increased in the 5/6 Nx groups compared with the sham-operated group (*n* = 10) at 7 weeks and 12 weeks; however, Ad-netrin-1 treatment (*n* = 10) had a significantly beneficial effect on reducing Scr and BUN levels (Fig. 1, *P* < 0.05). In H&E-stained images of kidney sections, the kidneys of the sham-operated rats showed no significant pathologic changes (Fig. 2, *n* = 10). However, focal interstitial fibrosis with tubular dilatation and shrinkage was observed in the vehicle-treated 5/6 Nx group. The pathological changes were alleviated in the Ad-netrin-1-treated 5/6 Nx group. Masson staining showed that the area of interstitial fibrosis in the vehicle-treated 5/6 Nx group was significantly greater than that in the sham-operated group. A smaller interstitial space was observed in the Ad-netrin-1-treated 5/6 Nx group. These data suggest that netrin-1 protected renal function in the 5/6 Nx rats.

### Netrin-1 attenuates EndoMT in 5/6 Nx rats

Previous studies have shown that EndoMT contributes to the accumulation of fibroblasts in tumors [22]. Here, we tested the hypothesis that EndoMT occurs in 5/6 Nx rat.

**Fig. 3:**
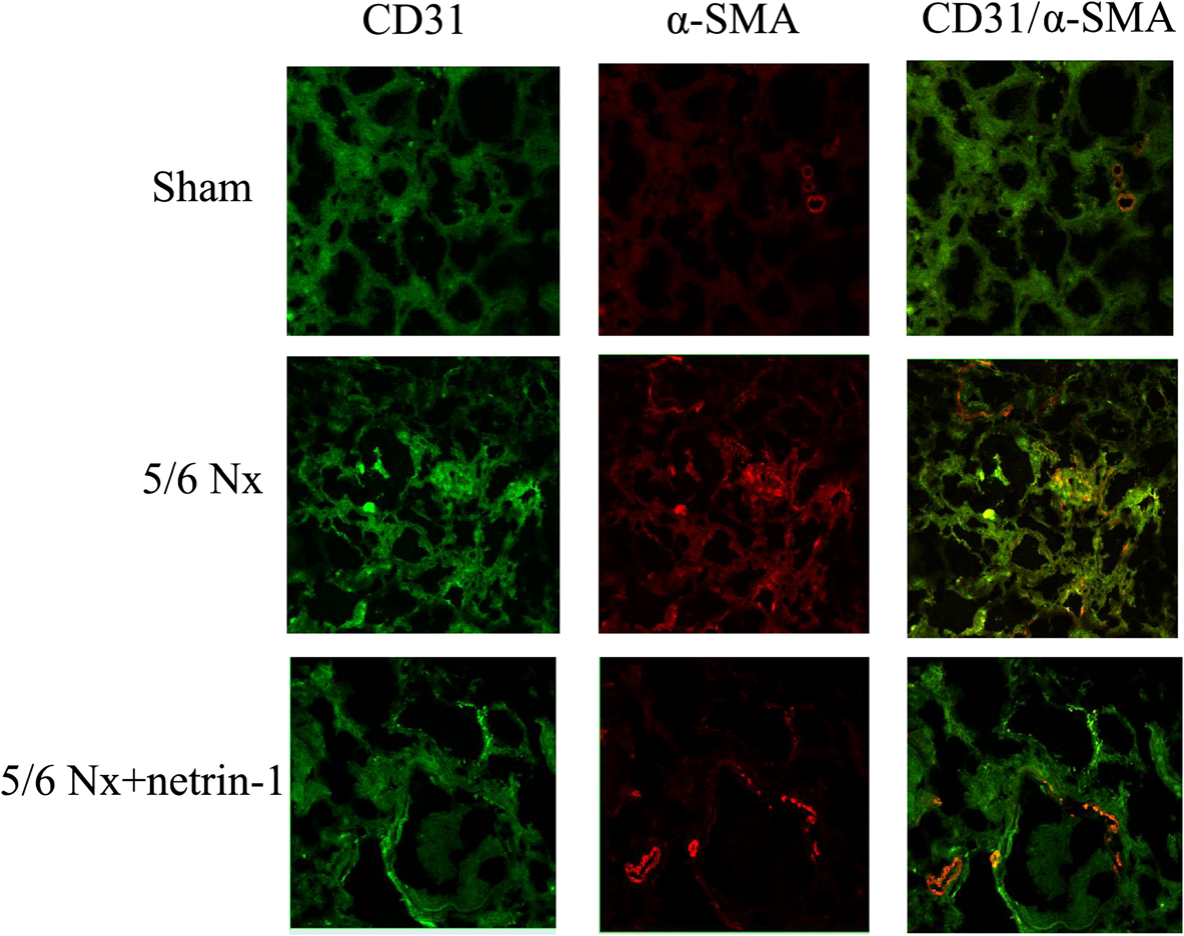
Netrin-1-induced inhibition of EndoMT in 5/6 Nx rats, as assessed by laser scanning confocal microscopy. Representative immunofluorescence images showing CD31 (green) and α-SMA (red) labeling. Double-positive renal cells were detected in the sham-operated group, and 5/6 Nx groups with and without Ad-netrin-1 treatment. The administration of netrin-1 reversed these effects (*P* < 0.05). The experiments were repeated three times

Renal tissues from the sacrificed rats were double-labeled using antibodies against the endothelial marker CD31 and the fibroblast marker α-SMA, which are indicators of EndoMT. Confocal microscopy revealed colocalization of CD31 and α-SMA (Fig. 3). Further, we observed that CD31 immunoreactivity decreased and some CD31-positive vessels acquired α-SMA staining in the vehicle-treated 5/6 Nx rats compared with sham-operated rats. α-SMA positive fibroblast populations were substantially increased in the 5/6 Nx rats compared with the sham-operated controls, which suggests that EndoMT had occurred in 5/6 Nx rats. The administration of Ad-netrin-1 markedly reduced the number of CD31+/α-SMA+ double-labeled cells in the Ad-netrin-1-treated 5/6 Nx rats. EndoMT involves a progressive loss of the endothelial cell marker CD31 associated with a reciprocal gain of the mesenchymal marker α-SMA. These findings suggest that EndoMT may account for a considerable portion of the fibroblasts in 5/6 Nx rats, and that EndoMT can be attenuated by netin-1.

### Netrin-1 affects the mRNA and protein expression of CD31, α-SMA and snail

**Fig. 4:**
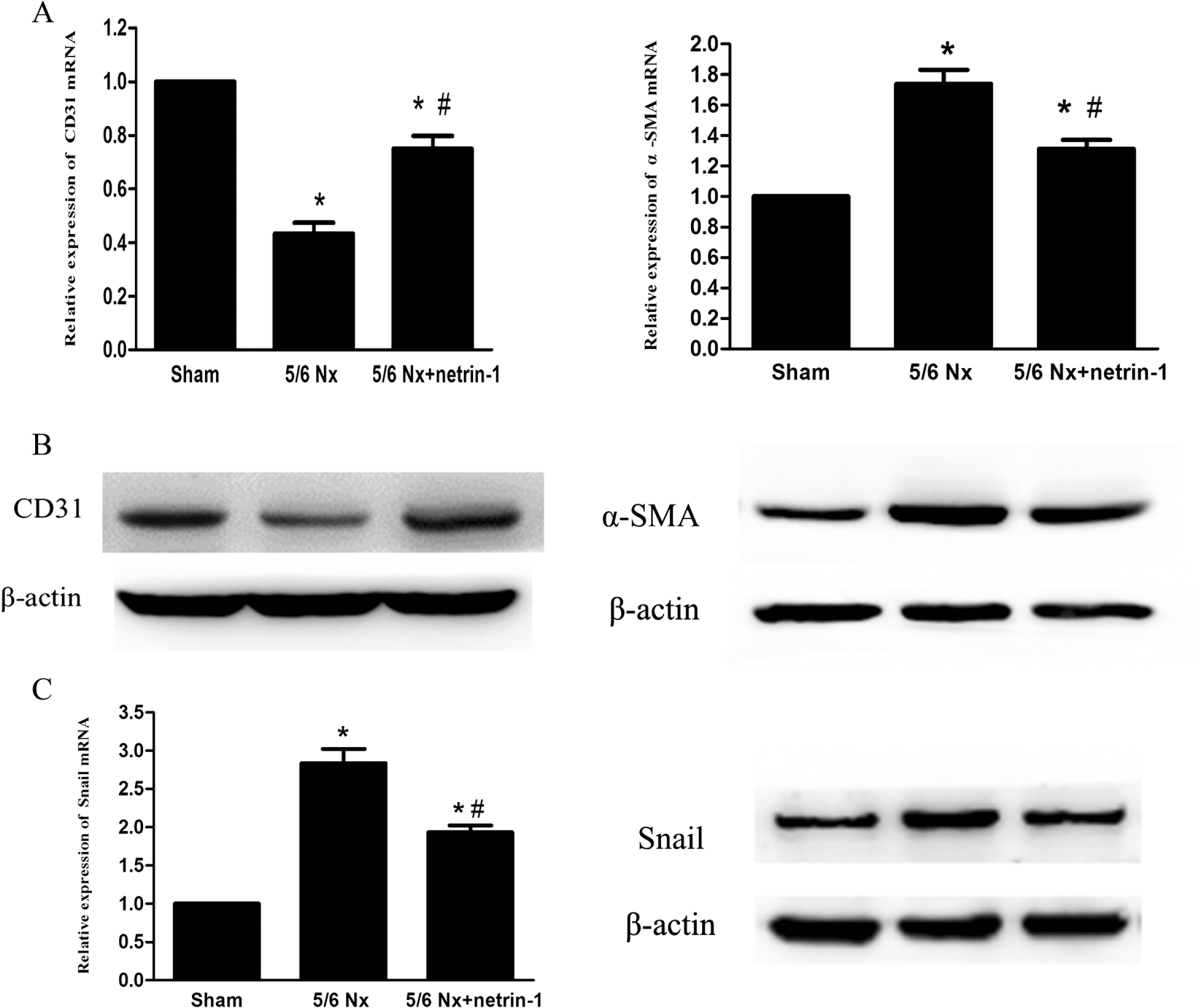
Expression of CD31, α-SMA and Snail in the kidney. **a** CD31 and α-SMA mRNA expression was quantified by real-time PCR and normalized to β-actin mRNA expression in the kidney tissues of sham-operated rats treated with the control adenovirus at postoperative week 13 (sham), 5/6 Nx rats treated with the control adenovirus (5/6 Nx), and 5/6 Nx rats treated with Ad-netrin-1 (5/6 Nx + netrin-1). **b** A representative western blot of the proteins extracted from the three experimental groups, indicating the level of CD31 and α-SMA protein expression relative to β-actin. **c** Relative expression of Snail mRNA was determined by realtime RT-PCR and normalized to β-actin mRNA (left panel). Snail protein levels were assessed by Western blot (right panel). All data are expressed as mean ± SEM; *, *P* < 0.05 vs. sham; #, *P* < 0.05 vs. 5/6 Nx

Next we investigated the expression levels of CD31 and α-SMA in renal tissues by Western blotting and real-time PCR. Mesenchymal mRNA and protein expression profiles showed increased expression of α-SMA and decreased expression of the endothelial marker CD31 in the vehicle-treated 5/6 Nx group compared with the sham-operated group (Fig. 4, *P* < 0.05). However, α-SMA expression was markedly decreased and CD31 expression was increased in the Ad-netrin-1-treated 5/6 Nx group compared with the vehicle-treated 5/6 Nx group. There are a number of key factors required for EndoMT. These include Snail and E-cadherin. Snail is a master regulator of EndoMT and can repress E-cadherin expression [23]. Snail levels were up-regulated in the 5/6 Nx rats. This was down-regulated following administration of netrin-1. These findings indicate disruption of EndoMT by Netrin-1 in 5/6 Nx rats.

### Netrin-1 affects TGF-β expression

**Fig. 5:**
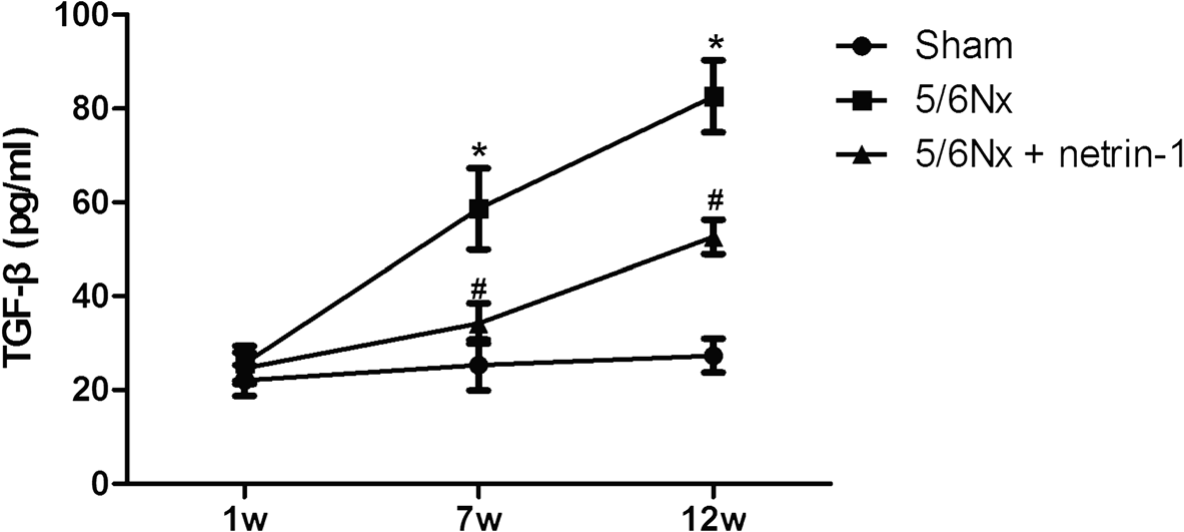
ELISA study to determine the concentration of TGF-β. Analyses were additionally performed at postsurgery weeks 7 and 12. Data are presented as mean ± SEM). *, *P* < 0.05 vs. sham; #, *P* < 0.05 vs. 5/6 Nx

TGF-β is a potent inducer of endothelial to mesenchymal transitions. In addition, it is important in EndoMT. We investigated the expression levels of TGF-β by ELISA. The serum of the vehicle-treated 5/6 Nx group showed stronger TGF-β expression compared to the sham-operated group; however, TGF-β expression in the Ad-netrin-1-treated 5/6 Nx group was significantly decreased compared with the vehicle-treated 5/6 Nx group (Fig. 5, *P* < 0.05). These findings indicate that Netrin-1 repressed TGF-β expression in the 5/6 Nx rats.

## Discussion

In our previous study [17], we reported that netrin-1 appears to be mediated by preservation of PTC endothelium and is associated with partial reversal of impaired angiogenesis, which reduce renal fibrosis in the 5/6 Nx rat model of kidney disease. Other work had demonstrated that EndoMT contributes to the early development of renal interstitial fibrosis in mice with streptozotocin (STZ)-induced diabetes [24]. Based on these findings, we concluded that netrin-1 plays an important role in EndoMT in 5/6 Nx rats. In the present study, using netrin-1-expressing adenovirus, we have provided evidence for the role of netrin-1 in EndoMT that occurs in CKD by showing that netrin-1 can prevent renal dysfunction and attenuate the process of EndoMT in the 5/6 Nx rat model. CKD is histologically characterized by interstitial fibrosis, which may be driven by peritubular capillary dropout and hypoxia. In kidney fibrosis, EndoMT is an important mechanism contributing to the accumulation of activated fibroblasts and myofibroblasts. In this study, we found evidence for potential EndoMT states in 5/6 Nx rats, which are a well-established model of CKD. EndoMT was assessed based on the expression of α-SMA in cells carrying the endothelial-specific cell surface marker CD-31 in the vehicle-treated 5/6 Nx rats. In support of this, it has been reported that co-expression of the endothelial cell marker CD31 and fibroblast marker α-SMA in the endothelium is indicative of an early stage of EndoMT [6].

Our study found that netrin-1 pretreatment resulted in blocking of EndoMT in 5/6 Nx rats; however, the mechanism via which netrin-1 attenuates renal fibrosis by blocking EndoMT remains largely unknown. Studies have demonstrated that TGF-β, bone morphogenic protein (BMP), and the Notch pathways are related to EndoMT [4, 6]. For example, EndoMT in CCM1-ablated endothelial cells is mediated by the activation of the TGF-β and BMP signaling pathways; moreover, inhibitors of the TGF-β and BMP pathways prevent EndoMT both in vitro and in vivo [25]. Further, the TGF-β pathway-induced inhibition of EndoMT reduced renal fibrosis and retarded the progression of nephropathy [26]. Studies have demonstrated that TGF-β stimulates EndMT through the Smad, p38 MAPK signalling pathways. These pathways are essential for increasing the expression of the cell-adhesion-suppressing transcription factor Snail [27]. Snail is best known for its ability to trigger EndMT. In this study, we found strong Snail expression in 5/6 Nx rats. However, Snail expression was much lower in the Ad-netrin-1-treated 5/6 Nx rats. Therefore, we propose that netrin-1 might inhibit EndoMT by blocking the TGF-β/Snail/α-SMA signaling pathway.

Recent studies report that netrin-1 protects the kidney against ischemia-reperfusion injury and attenuates diabetic kidney disease [28]. This indicates the protective role of the anti-inflammatory molecule netrin-1 in diabetic kidney disease; in particular, specific overexpression of netrin-1 in proximal tubular epithelial cells suppressed inflammation and albuminuria [29]. These findings suggest that netrin-1 suppresses kidney inflammation by inhibiting the cyclooxygenase-2 (COX-2)-mediated production of prostaglandin E2 (PGE2) in AKI and streptozotocin-induced diabetic mice [30]. Netrin-1 also suppressed AKI-induced tubular atrophy, interstitial fibrosis, vascular dropout, and glomerular sclerosis through suppression of STAT3 and JNK signaling and IL-6 expression in tubular epithelial cells [31]. Therefore, several pathways and factors may play a role in the anti-fibrotic effect of netrin-1. Based on our findings, further studies are needed to elucidate the signal pathway underlying the effect of netrin-1 in EndoMT-mediated protection of the kidney.

## Conclusions

EndoMT contributes to renal fibrosis in 5/6 Nx rats and can be prevented by netrin-1 treatment. These results imply that netrin-1 might exert its protective role in preventing CKD via an EndoMT-inhibiting mechanism. Netrin-1 may inhibit EndoMT by blocking the TGF-β/Snail/signaling pathway. Our results suggest that netrin-1 may represent a potential therapeutic agent for treating interstitial fibrosis. Further, the EndoMT-inhibiting mechanism in the kidney may also indicate its role in the prevention of renal disease progression.

### Ethics

All animal procedures were conducted according to the animal care and ethics laws and were approved by the Animal Care Committee of the General Hospital of Shenyang Military Area Command.

### Consent to participate

Not applicable, there is no patients participate within the study.

### Consent to publish

Not applicable.

### Availability of data and materials

All data underlying the findings are within the paper.

## Abbreviations

Ad-netrin-1: recombinant adenovirus expressing the netrin-1 gene
BMP: bone morphogenic protein
BUN: blood urea nitrogen
CKD: chronic kidney disease
COX-2: cyclooxygenase-2
EMT: epithelial-mesenchymal transition
EndoMT: endothelial-to-mesenchymal transition
Nx: 5/6nephrectomized
PFU: plaque-forming units
PGE2: production of prostaglandin E2
RT-PCR: real-time PCR
Scr: serum creatinine
SMCs: smooth muscle cells
TBS: Tris-buffered saline
TGF: Transforming growth factor
